# Harnessing the antibacterial activity of *Quercus infectoria* and *Phyllanthus emblica* against antibiotic-resistant *Salmonella* Typhi and *Salmonella* Enteritidis of poultry origin

**DOI:** 10.14202/vetworld.2020.1388-1396

**Published:** 2020-07-21

**Authors:** Amruta Nair, T. Balasaravanan, Sunil Jadhav, Vysakh Mohan, Chethan Kumar

**Affiliations:** 1 Department of Veterinary Public Health, Indian Veterinary Research Institute, Bareilly, Uttar Pradesh, India; 2 Department of Biotechnology, Nehru Arts and Science College, Coimbatore, Tamil Nadu, India; 3 Department of Animal Nutrition, Indian Veterinary Research Institute, Bareilly, Uttar Pradesh, India; 4 Department of Animal Science, Central Coastal Agricultural Research Institute, Goa, India

**Keywords:** antimicrobial-resistant, *Phyllanthusemblica*, phytochemicals, gas chromatography-mass spectrometry, *Quercusinfectoria*, *Salmonella*

## Abstract

**Background and Aim:**

In a scenario of the ineffectiveness of the current drugs against antibiotic-resistant pathogens, the herbal extracts can serve as an alternative remedy. This study appraises the antibacterial potency of *Quercus infectoria* (gall), *Phyllanthus emblica* (fruit) individually and synergistically against antimicrobial-resistant (AMR) *Salmonella* Typhi and *Salmonella* Enteritidis in a time and dose-dependent manner. Further, the antibacterial phytocompounds were identified employing gas chromatography-mass spectrometry (GC-MS).

**Materials and Methods:**

Preliminary antibacterial activity of the plant extracts was assessed using the agar disk diffusion method. *In vitro* evaluations of *Q. infectoria* methanolic extract (QIME) and *P. emblica* methanolic extract (PEME) against *S*. Typhi and *S*. Enteritidis were carried out using plate count method.

**Results:**

QIME and PEME at a dose rate of 50 mg/ml and 25 mg/ml, respectively, had a complete bactericidal effect on AMR *S*. Typhi and *S*. Enteritidis whereas 10 log_10_ CFU/ml of exponential growth was seen in untreated control groups. At the lower concentrations, QIME and PEME had a significant bacteriostatic effect (3-6 log_10_ reduction of the test isolates). The synergistic antibacterial effect obtained from the combination of these two plant extracts at 12.5 mg/ml was superior (p<0.001) than the individual treatments. Phytochemical profiling indicated the presence of tannins, flavonoids, saponins, and terpenoids in both the plant extracts. GC-MS analysis of QIME and PEME revealed the presence of 16 and 15 antibacterial phytocompounds, respectively. Further 1, 2, 3 Benzenetriol was found as the prominent active principle.

**Conclusion:**

The findings validate that QIME and PEME are potential antibacterial agents against AMR *S*. Typhi, *S*. Enteritidis and can play a promising role in antimicrobial packaging, poultry feed additives and can also serve as a platform for formulating effective phytotherapeutics.

## Introduction

*Salmonella* has coevolved and ensured continuous survival within humans by means of challenging the antibiotic regime and replicating tactfully in new hosts. *Salmonella* is one of the major contributors to the global public health burden with the highest incidence of 40% infection in infants and children under 5 years of age [1]. In India, the typhoidal and non-typhoidal form of salmonellosis is endemic and causes substantial morbidity and mortality in both pediatric and adult populations [2]. It is estimated that the incidence of culture-confirmed typhoid fever in India is 377/100,000 population per year [3] whereas, such estimates are lacking for non-typhoidal *Salmonella* [4]. The antimicrobial resistance (AMR) has been acknowledged as one of the primary threats to global health, food security, and socio-economic development worldwide. Earlier investigations have reported an increase in multidrug resistance among *Salmonella* Typhi strains in India [3]. Treatment success rates using antibiotics against *Salmonella* infections remain alarmingly poor with relapses, reinfections, and chronic carriages worsening the situation [5]. The indiscriminate use of antibiotics in clinical practices and food industry as growth promoters in livestock feed is the prime stimulus to elicit a bacterial adaptation response causing AMR. Recently, retail chicken meat has emerged, as a potential source of antibiotic-resistant *Salmonella* in the food chain [6]. Hence, any prophylactic measure to curb salmonellosis in humans must take into account the role of poultry in the dissemination of AMR *Salmonella*.

The usage of plant extracts to combat life-threatening infections and for therapeutic purposes dates back to antiquity. Of late in developing countries, the use of plant extracts has gained momentum over antibiotics because of their availability, biodegradability, utilization in the pharmaceutical, and food industry [7]. In this context, several research reports have documented the antimicrobial activity of various plant extracts against several Gram-positive and Gram-negative pathogens [8]. *Quercus infectoria* is known to possess medicinal properties such as astringent, anti-inflammatory, antimicrobial, antidiabetic, and gastroprotective activities [9]. Similarly, *Phyllanthus emblica* has antidiabetic, hypolipidemic, antibacterial, and antioxidant properties [10]. It is used in traditional Indian medicine for the treatment of diarrhea and jaundice.

Nevertheless, there is a paucity of comprehensive analysis of the antibacterial activities of these plant extracts utilizing gas chromatography-mass spectrometry (GC-MS) phytochemical profiling against foodborne zoonotic pathogens. Hence, the goal of this study was to assess the antibacterial activity of these plant extracts individually and synergistically against antibiotic-resistant *S*. Typhi and *Salmonella* Enteritidis of poultry origin and to identify the putative bioactive agents responsible for the antibacterial activity.

## Materials and Methods

### Ethical approval and informed consent

All the procedures have been carried out in accordance with the relevant national and institutional guidelines laid down by the Institutional Ethics Committee. The poultry meat and droppings samples were collected from organized or unorganized farms after having proper consent from animal owners.

### Study period and location

Plant specimens were collected between February 2016 and September 2016 from Palani Hills, Western Ghats, Tamil Nadu, India. Preliminary antimicrobial activity assessment, phytochemical analysis, *in vitro* experiments and GC-MS analysis were completed in February 2018 at Nehru Arts and Science College, Coimbatore.

### Bacterial strains

A total of 25 field isolates of *S*. Enteritidis (n=14) and *S*. Typhi (n=11) recovered from poultry meat and poultry droppings were included in this study. All the isolates were subjected to recommended tests described in the FDA bacteriological analytical manual [11] and further multiplex polymerase chain reaction assays for serotype confirmation [12].

### Antimicrobial susceptibility screening employing commercial disks

The antimicrobial susceptibility test for all the *Salmonella* isolates was performed and interpreted according to the Clinical Laboratory Standards Institute (CLSI) guidelines, using disk diffusion assay. *Escherichia coli* ATCC 25922 was used as a quality control strain [13]. A total of nine antimicrobial disks (HiMedia, India) comprising ampicillin (10 μg), ceftriaxone (30 μg), tetracycline (30 μg), ciprofloxacin (10 μg), co-trimoxazole (25 μg), imipenem (10 μg), nalidixic acid (30 μg), sulfisoxazole (50 μg), and chloramphenicol (30 μg) were tested. The zone of inhibition (diameter in mm) for each antibiotic was initially measured for the quality control strain, followed by all the test strains (Supplement Table-1). The antimicrobial susceptibility assay was performed in triplicate and the data obtained were compared with CLSI interpretative chart.

### Plant extracts preparations

*Q. infectoria* (galls) and *P. emblica* (fruit) were collected from Palani Hills, Western Ghats, Tamil Nadu. The plant specimens were identified at the Government Arts College, Ariyalur, Tamil Nadu. The ethanolic, methanolic, and aqueous extracts of Q. *infectoria* and P. *emblica* were prepared and resolved in 2.5% of dimethyl sulfoxide (DMSO) as described previously [14]. In brief, the *Q. infectoria* galls and *P. emblica* fruits were washed under tap water and dried at room temperature. The dried plant material was ground into fine particles and was weighed and soaked in absolute ethanol, methanol, and autoclaved double distilled water at the ratio of 1:20 (w/v) for 48 h. Extracts were then filtered through Whatman No. 1 filter paper to separate the plant residues, followed by rotary evaporation to remove the excess ethanol and methanol and obtain crude extracts. The crude extracts were kept at 4°C until further use.

### Preliminary screening of various solvent based extractives of *Q. infectoria* and *P. emblica* for antibacterial activity

The preliminary antibacterial activity of *Q*. *infectoria* and *P*. *emblica* extract against *S*. Typhi and *S*. Enteritidis was determined following the methodology proposed by the previous studies [15] with minor modification. In brief, the sterile filter paper disks (6 mm diameter) were individually impregnated with 30 μl of DMSO resolved aqueous, ethanolic, and methanolic extracts of *Q. infectoria* and *P. emblica*, respectively, were placed on Petri plates seeded (10^8^ CFU/ml) with AMR *S*. Typhi and *S*. Enteritidis, ciprofloxacin (10 μg) as sensitive control, and nalidixic acid (30 μg) as resistant control. These inoculated Petri plates were then incubated at 37°C for 24 h. The diameter of the zone of inhibition (>9 mm) was recorded. Each experiment was carried out in triplicate and the average was recorded.

### Individual *in vitro* antibacterial screening of *Q. infectoria* and *P. emblica* against AMR *S.* Typhi and *S.* Enteritidis at different time intervals

The desired log phase cultures of *S*. Typhi and *S*. Enteritidis were individually enumerated using standard 0.5 McFarland standard tubes. Then, the cultures were resuspended in Dulbecco’s modified eagle’s medium (DMEM) of pH 7.2. Subsequently, to evaluate the antibacterial activity of *Q. infectoria* methanolic extract (QIME) and *P. emblica* methanolic extract (PEME) individually against AMR *S*. Typhi, the following groups were formed:

- Group 1: 1×10^7^ CFU of resistant *S*. Typhi + 50 mg/ml QIME (Q1) or 50mg/ml PEME (P1)
- Group 2: 1×10^7^ CFU of resistant *S*. Typhi + 25 mg/ml QIME (Q2) or 25 mg/ml PEME (P2)
- Group 3: 1×10^7^ CFU of resistant *S*. Typhi + 12.5 mg/ml QIME (Q3) or 12.5 mg/ml PEME (P3)
- Group 4: 1×10^7^ CFU of resistant *S*. Typhi + 6.25 mg/ml QIME (Q4) or 6.25 mg/ml PEME (P4)
- Group 5: 1×10^7^ CFU of resistant *S*. Typhi + 3.12 mg/ml QIME (Q5) or 3.12 mg/ml PEME (P5)
- Group 6: 1×10^7^ CFU of resistant *S*. Typhi + 3.12 mg/ml Ciprofloxacin (Treatment Control)
- Group 7: 1×10^7^ CFU of resistant *S*. Typhi + DMEM (Untreated control)
- Group 8: 1×10^7^ CFU of resistant S. Typhi + 2.5% DMSO (Untreated control)

Similar experimental groups were formed and investigated to appraise the antibacterial activity of QIME and PEME against AMR *S*. Enteritidis. All the groups of *S*. Typhi and *S*. Enteritidis with various concentrations of plant extracts were incubated at 37°C and were assessed for 72 h. To enumerate the antibacterial effect an aliquot of 100 μl was withdrawn from all the formed groups at 24, 48, and 72 h, respectively. All the aliquots were serially diluted ten-fold times in normal saline solution up to a dilution of 10^-5^. From all the ten-fold serial dilutions, 10 μl was placed on nutrient agar (NA) plates and incubated at 37°C for 24 h [16].

### Synergistic *in vitro* screening of QIME+PEME against AMR strain of *Salmonella*

Briefly, to evaluate the synergistic antibacterial activity of QIME and PEME against resistant strain of *S*. Typhi, the following groups were formed:

- Group 1: 1×10^7^ CFU of resistant *S*. Typhi + 50 mg/ml QIME + 50 mg/ml PEME (M1)
- Group 2: 1×10^7^ CFU of resistant *S*. Typhi + 25 mg/ml QIME + 25 mg/ml PEME (M2)
- Group 3: 1×10^7^ CFU of resistant *S*. Typhi + 12.5 mg/ml QIME + 12.5 mg/ml PEME (M3)
- Group 4: 1×10^7^ CFU of resistant *S*. Typhi + 6.25 mg/ml QIME + 6.25 mg/ml PEME (M4)
- Group 5: 1×10^7^ CFU of resistant *S*. Typhi + 3.125 mg/ml QIME + 3.125 mg/ml PEME (M5)
- Group 6: 1×10^7^ CFU of resistant *S*. Typhi + 3.125 mg/ml Ciprofloxacin (Treatment Control)
- Group 7: 1×10^7^ CFU of resistant *S*. Typhi + DMEM (Untreated control)
- Group 8: 1×10^7^ CFU of resistant *S*. Typhi + 2.5% DMSO (Untreated control)

Similarly, as aforesaid an analogous procedure was adopted to evaluate the synergistic antibacterial activity of *Q*. *infectoria* and *P*. *emblica* against AMR *S*. Enteritidis.

### Phytochemical profiling

The QIME and PEME were subjected to preliminary phytochemical screening and were carried out using the standard procedure described in the earlier study [17].

### GC-MS analysis

The QIME and PEME were further subjected to GC-MS to quantify and identify bioactive compounds. Briefly, 2 μl of the sample was injected into a fission GC8000 series GC coupled to a MD800 MS with quadrupole mass analyzer (Fission instrument, Milano, Italy) and DB5-MS column. The injector temperature was 230°C. Helium was used as the carrier gas at a constant flow rate of 1mL/min. The oven temperature was maintained as described in earlier studies [18]. Mass spectra of phytocompounds were compared with the database of the National Institute of Standard and Technology.

### Statistical analysis

All the *in vitro* experiments were performed in triplicate and the values were expressed as mean ± standard deviation. The level of significance between treatment groups and controls was analyzed by Student’s *t*-test, two-way ANOVA followed by Dunnett’s multiple comparison test. p≤0.05 was considered as significant and p≤0.001 as highly significant.

## Results

### Antibiogram of *S.* Typhi and *S.* Enteritidis isolates using disk diffusion assay

Based on the outcome of the antibiogram profile (Table-1), out of the 25 *Salmonella* isolates only two *Salmonella* strains (*S*. Enteritidis and *S*. Typhi) were found to be antibi­otic-resistant. *S*. Enteritidis was resistant to ampicillin, nalidixic acid, and sulfisoxazole while *S*. Typhi was resistant to nalidixic acid, sulfisoxazole, and ceftriaxone. These strains were used to evaluate the antibacterial activity of plant extracts.

**Table-1:** Susceptibility profile of antibiotic resistant strains of *Salmonella.*

Antibiotic	Concentration	*Salmonella* isolate resistant/intermediate/sensitive	

		*S.* Typhi	*S.* Enteritidis
Chloramphenicol	30 μg	S	S
Ciprofloxacin	10 μg	S	S
Ampicillin	10 μg	S	R
Tetracycline	30 μg	S	S
Ceftriaxone	30 μg	R	S
Nalidixic acid	30 μg	R	R
Sulfisoxazole	50 μg	R	R
Imipenem	10 μg	S	S
Co-trimoxazole	25 μg	S	S

*S.* Typhi=*Salmonella* Typhi, *S*. Enteritidis=*Salmonella* Enteritidis

### Preliminary antibacterial efficacy of *Q. infectoria* and *P. emblica* against AMR *S.* Typhi and *S.* Enteritidis

The analysis of preliminary antibacterial activity validated that methanol extract of both the plants had significant and higher antimicrobial activity than the ethanol and aqueous extract against *S*. Typhi and *S*. Enteritidis, respectively (Table-2).

**Table-2:** Antibacterial profiles of *Q. infectoria* extract and *P. emblica* extract using different solvents and respective controls.

Solvent	Diameter of inhibition zone (mm) ± SD			

	*Q. infectoria* extract		*P. emblica* extract	
	
	*S*. Typhi	*S*. Enteritidis	*S*. Typhi	*S*. Enteritidis
Ethanol	13.4±0.3	14.2 ±0.5	15.6±0.6	12.4±0.6
Methanol	15.4±0.2	16.5±0.7	16.2±0.4	15.6±0.5
Distilled water	11.3±0.6	14.0 ±0.3	12.2±0.5	10.2±0.4
DMSO (−control)	0.0±0.0	0.0±0.0	0.0±0.0	0.0 ±0.5
Ciprofloxacin (+control)	25.5±0.7	26.0±0.5	26.0±0.3	26.5±0.5
Nalidixic acid (−control)	06.0±0.4	05.0±0.3	06.0±0.4	05.0±0.8

The antibacterial effects of plant extracts were considered to be statistically significant at p≤0.05. *Q. infectoria*=*Quercus infectoria, P. emblica*=*Phyllanthus emblica, S.* Typhi=*Salmonella* Typhi, *S*. Enteritidis=*Salmonella* Enteritidis

### Antibacterial effect of QIME against AMR *S.* Typhi and *S.* Enteritidis

In Group 1, a highly significant antibacterial activity of QIME at a concentration of 50 mg/ml was evident wherein complete inhibition of *S*. Typhi was observed at all the analyzed time points. However, their respective DMEM and DMSO untreated controls revealed a growth of 10 log10 CFU/ml on NA (Table-3). In Group 2, a substantial 4 log reduction at 24, 48 h, and 6 log reduction in the bacterial count at 72 h was observed when compared with control.

**Table-3:** Dose and time-dependent bactericidal effects of different herbal extracts on *S.* Typhi and *S.* Enteritidis.

Groups	Period (h)		

	24	48	72
*Q. infectoria* against *S.* Typhi			
Q1 (50 mg)	ND	ND	ND
Q2 (25 mg)	6.50±0.31	6.56±0.30	4.55±0.32
Q3 (12.5 mg)	7.52±0.37	7.78±0.23	7.43±0.22
Q4 (6.25 mg)	7.60±0.42	7.58±0.36	7.53±0.41
Q5 (3.125 mg)	7.66±0.34	7.58±0.31	7.50±0.29
CIP control	ND	ND	ND
DMEM	10.64±0.33	10.60±0.24	10.72±0.36
DMSO control	10.52±0.41	10.59±0.23	10.57±0.26
*P. emblica* against *S.* Typhi			
P1 (50 mg)	ND	ND	ND
P2 (25 mg)	ND	ND	ND
P3 (12.5 mg)	7.53±0.30	6.47±0.40	5.63±0.16
P4 (6.25 mg)	7.43±0.20	7.41±0.27	6.50±0.32
P5 (3.125 mg)	7.47±0.24	7.49±0.48	6.49±0.34
CIP control	ND	ND	ND
DMEM control	10.41±0.34	10.58±0.17	10.55±0.29
DMSO CONTROL	10.42±0.35	10.56±0.18	10.73±0.23
*Q. infectoria + P. emblica* against *S*. Typhi			
M1 (50 mg)	ND	ND	ND
M2 (25 mg)	ND	ND	ND
M3 (12.5 mg)	ND	ND	ND
M4 (6.25 mg)	7.46±0.29	6.58±0.33	5.59±0.19
M5 (3.125 mg)	7.54±0.30	6.73±0.21	6.38±0.19
CIP control	ND	ND	ND
DMEM control	10.38±0.34	10.62±0.26	10.75±0.18
DMSO control	10.29±0.23	10.58±0.24	10.65±0.16
*Q. infectoria* against * S. Enteritidis*			
Q1 (50 mg)	ND	ND	ND
Q2 (25 mg)	6.47±0.29	5.61±0.35	3.87±0.70
Q3 (12.5 mg)	7.52±0.33	7.45±0.36	7.34±0.37
Q4 (6.25 mg)	7.68±0.26	7.58±0.33	7.52±0.24
Q5 (3.125 mg)	7.70±0.24	7.55±0.23	7.55±0.24
CIP control	ND	ND	ND
DMEM control	10.54±0.30	10.60±0.24	10.72±0.36
DMSO control	10.52±0.19	10.79±0.16	10.55±0.34
*P. emblica* against *S. Enteritidis*			
P1 (50 mg)	ND	ND	ND
P2 (25 mg)	ND	ND	ND
P3 (12.5 mg)	7.74±0.22	7.49±0.28	4.58±0.22
P4 (6.25 mg)	7.56±0.32	7.52±0.26	7.32±0.22
P5 (3.125 mg)	7.66±0.28	7.58±0.28	7.51±0.29
CIP control	ND	ND	ND
DMEM control	10.64±0.33	10.78±0.19	10.64±0.28
DMSO control	10.59±0.24	10.72±0.19	10.58±0.32
*Q. infectoria + P. emblica* against *S. Enteritidis*			
M1 (50 mg)	ND	ND	ND
M2 (25 mg)	ND	ND	ND
M3 (12.5 mg)	ND	ND	ND
M4 (6.25 mg)	6.65±0.30	6.53±0.22	4.62±0.20
M5 (3.125 mg)	7.67±0.45	7.48±0.18	7.35±0.31
CIP control	ND	ND	ND
DMEM control	10.44±0.39	10.53±0.44	10.54±0.37
DMSO control	10.66±0.29	10.46±0.29	10.55±0.34

ND=Not detected. The antibacterial effects were considered to be statistically highly significant at p<0.001. S. Typhi=*Salmonella* Typhi, S. Enteritidis=*Salmonella* Enteritidis, Q. infectoria=Quercus infectoria, *P. emblica*=*Phyllanthus emblica*, DMSO=Dimethyl sulfoxide, DMEM=Dulbecco’s modified eagle’s medium, CIP=Ciprofloxacin

Similarly, the antibacterial effects of QIME on the growth and survival of *S*. Enteritidis over a period of 24-72 h were ascertained with appropriate controls. The bactericidal response was highly significant in Group 1 following exposure to 50 mg/ml of QIME at all the designated time points whereas, the respective untreated controls (Group 7-Group 8) revealed the growth of about 10 log10 CFU/ml on NA plates at designated time intervals (Table-3). At a dose of 25 mg (Group 2), a systematic and noteworthy antibacterial effect was observed in bacterial growth displaying 4 log reduction at 24 h, 5 log reduction at 48 h, and 7 log reduction in the bacterial count at 72 h which was observed (Table-3). QIME exhibited a dose and time-dependent inhibition of *S*. Typhi and *S*. Enteritidis.

### Antibacterial effect of PEME against *S.* Typhi and *S.* Enteritidis

The data of the growth profile of *S*. Typhi co-incubated with various concentration of PEME at designated time points, along with appropriate controls are presented in Table-3. PEME exhibited a remarkable dose and time-dependent complete inhibition of *S*. Typhi at a concentration of 50 mg and 25 mg in Group 1 and Group 2, respectively. Contrastingly, an exponential growth 10 log 10 CFU/ml was recorded in the control group. In Group 3, a 3 log reduction at 24 h, 4 log reduction at 48 h, and 5 log reduction in the bacterial count at 72 h were observed (Table-3).

As stated above, in a similar mode, the antibacterial potency of PEME was assessed against *S*. Enteritidis using appropriate controls (Table-3) and more or less in Groups 1 and 2 similar kinds of highly significant 100% bactericidal effect were observed at a concentration 50 mg and 25 mg of PEME for *S*. Enteritidis. In Group 3, a 3 log reduction in the bacterial count was noticed at 24 h and 48 h post co-incubation whereas a drastic 6 log reduction was seen at 72 h post-co-incubation when compared with their respective control.

### Synergistic antimicrobial effect of the combination of QIME and PEME against *S.* Typhi and *S.* Enteritidis

The synergistic antimicrobial effects of QIME and PEME against AMR *S*. Typhi and *S*. Enteritidis are presented in Table-3. Highly pronounced bactericidal effect was observed against both *S*. Typhi and *S*. Enteritidis in Group 1, Group 2, and Group 3 at all the analyzed time points from dose rate ranging 50 mg/ml to a dose rate as low as 12.5 mg/ml when QIME and PEME were used in combination. The combination of QIME and PEME had a more profound antibacterial effect against *S*. Enteritidis in Group 4, wherein 4 log reduction in bacterial growth was achieved at 24 h and 48 h, respectively, followed by 6 log reduction at 72 h.

### Phytochemical analysis

On phytochemical screening, QIME revealed the presence of tannins, cardiac glycosides, phenols, steroids, flavonoids, terpenoids, and saponins whereas PEME with the exception of cardiac glycosides revealed the presence of all the aforesaid phytochemicals and alkaloids.

### GC-MS analysis of QIME and PEME

**Figure-1:**
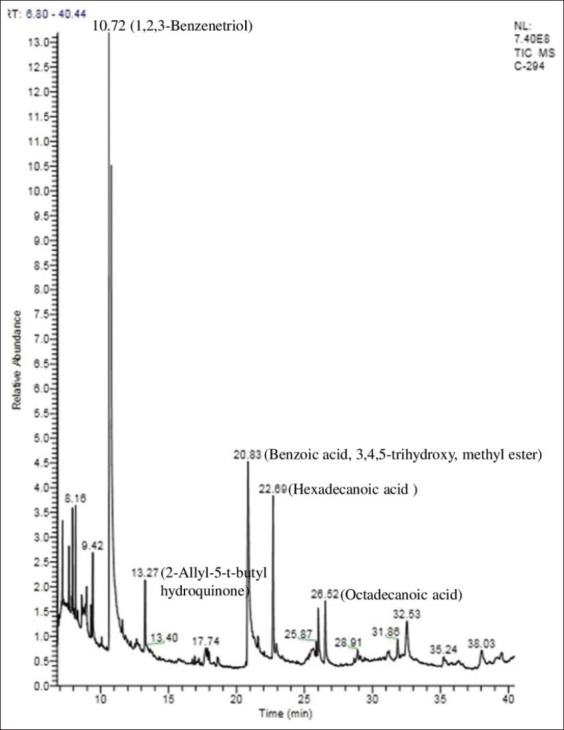
Chromatogram of methanol extract of *Quercus infectoria* galls by gas chromatography-mass spectrometry analysis.

**Figure-2:**
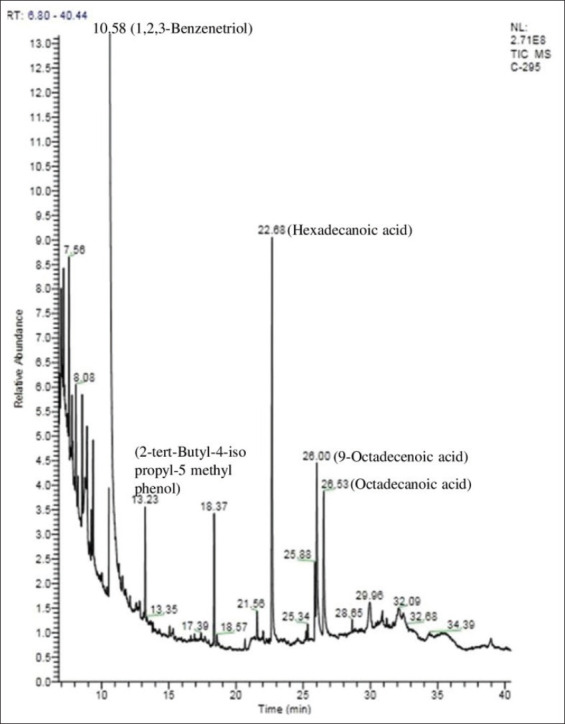
Chromatogram of methanol extract of *Phyllanthus emblica* fruits by gas chromatography-mass spectrometry analysis.

In virtue of retention time, molecular weight, peak height observed in the gas chromatography-mass spectrometry (GC-MS) chromatogram of QIME, approximately 23 phytocompounds were detected (Figure-1). Further, in this study based on NCBI PUB-CHEM analysis and literature survey, 16 active principles possessing antibacterial activity were identified in QIME (Supplement Table-2). Similarly, the GC-MS chromatogram of PEME revealed the presence of 18 phytocompounds (Figure-2). Among these, a total of 15 bioactive phytocompounds were found to have the antimicrobial property (Supplement Table-3). In the course of the present investigation on comparison of the GC-MS data a few of the secondary metabolites, namely, 1,2,3 Benzenetriol, hexadecanoic acid, 9-octadecenoic acid, octadecenoic acid, tetratetracontane, and nonacosane were found to be common in both QIME and PEME.

## Discussion

Antibiotic resistance is a multifactorial impediment. It is exacerbated by the lack of innovative therapeutic options in the current treatment strategy against pathogens of public health importance. This debacle has necessitated the exploration of novel, inexpensive, natural remedies to combat AMR. The antibiotic resistance pattern seen in this investigation is consistent with the previous reports from India, which has indicated a higher incidence rate of nalidixic acid resistance and the highest level of susceptibility toward ciprofloxacin in *S*. Enteritidis and *S*. Typhi [19,20]. Many strategies such as curbing the irrational antibiotic use in animals, enhanced surveillance and response systems and standard antibiotic policy have been implemented in India for the containment of AMR [21]. In this study, the antibacterial activity of the methanolic extractives of *Q*. *infectoria* and *P. emblica* was found to be higher than the aqueous and ethanol extracts, which is in concordance with the previous studies which have established the superiority of methanol [22]. Methanol is considered as the most reliable solvent for the extraction of antimicrobial phytocompounds from medicinal plants compared to other solvents [22]. Individually, the antimicrobial activity of *P*. *emblica* and *Q. infectoria* has been explored against *S*. Typhi, *Salmonella* Paratyphi, *Salmonella* Typhimurium, and other *Salmonella* spp. [9,23]. Most of the previous studies have explored the antibacterial activity of various plant extracts only up to a pilot level, employing a broth dilution method or disk diffusion assay. However, in this study, the extent of *in vitro* antibacterial activity of QIME and PEME has been assessed both individually and in combination, against AMR *S*. Enteritidis and *S*. Typhi of poultry origin, for up to 72 h post-treatment following the methodology of bacterial enumeration and plate count. In this study, 2.5% DMSO was used to resolve the QIME and PEME because earlier studies have observed that DMSO at a concentration of 4% and above was found to have an inhibitory effect on bacterial growth [24].

Similar to earlier studies in our study, dose-dependent inhibition was observed for *Q*. *infectoria* and *P*. *emblica* against *S*. Typhi and *S*. Enteritidis [25]. Precisely at a dose rate of 50 mg/ml of QIME, 25 mg/ml of PEME, and 12.5 mg/ml of combination had 100% bactericidal effect and completely inhibited *S*. Typhi and *S*. Enteritidis at all the analyzed time points. This study differs from other studies in terms of the methodology followed [16] and the combination used to evaluate the antibacterial activity and the extent to which the antibacterial activity was assessed. A similar kind of study employing combination of these two plant extracts has not been attempted earlier against AMR *Salmonella* of poultry origin. Basri and Fan [9] have reported that the growth of *S*. Typhimurium was inhibited by acetone extracts of *Q*. *infectoria* at MBC value of 1.25 mg/ml which is on the lower side than our results. With regard to the antimicrobial effect of *P*. *emblica*, our findings differ from the results of the previous studies wherein the methanolic extract of *P*. *emblica* exhibited a lower MBC against *S*. Typhi (MBC= 100 μg/ml) and *S*. Paratyphi (MBC= 25 μg/ml) [23]. A possible explanation for this variation can be attributed to the differences in the methodologies and specific strain used, type or part of plant extract investigated, the degree of solubility of the extracting solvent and also the agro-climatic conditions of the plant [26].

In concordance with the findings of the synergistic antibacterial effect seen in this study, it has been previously reported that equal proportions of the combination of different phytocompounds even those present in low amount, makes the preparation an exceptionally rich source of bioactive constituents and enhancing their activity [27].

Furthermore, the plant extracts were subjected to GC-MS analysis for precise information on the active compound. GC-MS is considered as the benchmark technique for the identification of phytocompounds. Our results are in consonance with the previous report, which confirms the effective antimicrobial activity of phenols, tannins. Tannins can damage bacterial membranes and hinder the matrix production, adhesion hence preventing the bacterial growth and biofilm production [28]. 1,2,3 Benzenetriol, the putative active principle found in this study is known to alter microbial cell permeability and produces reactive oxygen species [29]. Alkaloids are potential inhibitors of bacterial nucleic acid synthesis and cell division [30]. Fatty acids such as hexadecanoic acid, oleic acid bring about changes in the integrity of cell membrane compositions leading to cell swelling, cytoplasmic membrane damage, distension, and lysis [31]. Flavonoids can inhibit DNA and RNA synthesis in bacteria by means of intercalation or formation of hydrogen bonds, stacking of nucleic acid bases and thereby influencing the DNA gyrase activity [32,33].

It is worth mentioning that the phytocompounds embroil several chemical groups in their structure; hence their antimicrobial activity cannot be accredited to one specific mechanism [34]. All the five common phytocompounds of *Q*. *infectoria* and *P*. *emblica* have been individually analyzed for their antibacterial activity against various pathogens by several researchers (Supplement Tables-2 and 3). However, most of the above-mentioned compounds so far have not been analyzed for their antibacterial activity against *S*. Enteritidis.

In contrast to commercial antibiotics, the possibility of the bacteria developing resistance mechanism toward plant-derived antimicrobials is trivial [34]. Many food applications were reported for *Q*. *infectoria* powders and extract formulations. These extracts are generally recognized as safe which reinforces their potential biosafety human uses [35]. Several investigators have reported strong anti-quorum sensing activity and anti-biofilm activity of *Q*. *infectoria* and *P*. *emblica* against bacterial pathogens [36]. Hence, QIME and PEME can be used in poultry feed as a judicious alternative to combat *Salmonella* infection and limiting its dissemination. QIME and PEME can serve as prospective antimicrobial packaging film or spray which can extend the shelf-life, improve the safety of food products.

## Conclusion

Overall, the findings of the present study emboldens the use of the methanolic extracts of *Q*. *infectoria* and *P*. *emblica* as an effective herbal antibacterial remedy to combat antibiotic-resistant *Salmonella*. The presence of bioactive phytocompounds, especially 1, 2, 3 Benzenetriol in QIME and PEME elicits a highly pronounced bactericidal effect at a higher dosage and bacteriostatic effect at a lower dosage against both AMR *S*. Typhi and *S*. Enteritidis. The combination of QIME and PEME can play a promising role in antimicrobial packaging and poultry feed. However, an *in vivo* trial in poultry is recommended to decide appropriate dose and efficacy. Moreover, from a future perspective, the phytocompounds identified in this study need to be further purified and studied to serve as a platform for formulating effective phytotherapeutics.

### Data availability

Supplementary data can be available from the corresponding author.

## Authors’ Contributions

AN and TB conceived the experimental design. AN and VM performed the experiments. SJ and CK participated in the phytochemical analysis of the plant extracts. AN, SJ, and TB analyzed the data. AN and TB drafted and revised the manuscript. All the authors have read and approved the final manuscript.
